# A *dietary carbohydrate – gut Parasutterella – human fatty acid biosynthesis* metabolic axis in obesity and type 2 diabetes

**DOI:** 10.1080/19490976.2022.2057778

**Published:** 2022-04-18

**Authors:** Lea Henneke, Kristina Schlicht, Nadia A. Andreani, Tim Hollstein, Tobias Demetrowitsch, Carina Knappe, Katharina Hartmann, Julia Jensen-Kroll, Nathalie Rohmann, Daniela Pohlschneider, Corinna Geisler, Dominik M. Schulte, Ute Settgast, Kathrin Türk, Johannes Zimmermann, Christoph Kaleta, John F. Baines, Jane Shearer, Shrushti Shah, Grace Shen-Tu, Karin Schwarz, Andre Franke, Stefan Schreiber, Matthias Laudes

**Affiliations:** a Institute of Diabetes and Clinical Metabolic Research, University of Kiel, Kiel, Germany; b Section of Evolutionary Medicine, Institute for Experimental Medicine University of Kiel, Kiel, Germany; c Guest group for evolutionary medicine Max-Planck-Institute of Evolutionary Biology, Plön, Germany; d Division of Food Technology, Department of Human Nutrition, University of Kiel, Kiel, Germany; e Research Group Medical System Biology, Institute of Experimental Medicine, University of Kiel, Kiel, Germany; f Department of Biochemistry and Molecular Biology, Cumming School of Medicine, Faculty Kinesiology, University of Calgary, Calgary, Alberta, Canada; g Alberta’s Tomorrow Project, Cancer Control Alberta, Alberta Health Services, Edmonton, AB, Canada; h Institute of Clinical Molecular Biology, Kiel University, Kiel, Germany; i Division of Endocrinology, Diabetes and Clinical Nutrition, Department of Medicine 1, University Medical Centre Schleswig-Holstein, Kiel University, Kiel, Germany

**Keywords:** Gut microbiome, L-cysteine, obesity, *Parasutterella*, fatty acid biosynthesis

## Abstract

Recent rodent microbiome experiments suggest that besides *Akkermansia, Parasutterella sp*. are important in type 2 diabetes and obesity development. In the present translational human study, we aimed to characterize *Parasutterella* in our European cross-sectional FoCus cohort (n = 1,544) followed by validation of the major results in an independent Canadian cohort (n = 438). In addition, we examined *Parasutterella* abundance in response to a weight loss intervention (n = 55). *Parasutterella* was positively associated with BMI and type 2 diabetes independently of the reduced microbiome α/β diversity and low-grade inflammation commonly found in obesity. Nutritional analysis revealed a positive association with the dietary intake of carbohydrates but not with fat or protein consumption. Out of 126 serum metabolites differentially detectable by untargeted HPLC-based MS-metabolomics, L-cysteine showed the strongest reduction in subjects with high *Parasutterella* abundance. This is of interest, since *Parasutterella* is a known high L-cysteine consumer and L-cysteine is known to improve blood glucose levels in rodents. Furthermore, metabolic network enrichment analysis identified an association of high *Parasutterella* abundance with the activation of the human fatty acid biosynthesis pathway suggesting a mechanism for body weight gain. This is supported by a significant reduction of the *Parasutterella* abundance during our weight loss intervention. Together, these data indicate a role for *Parasutterella* in human type 2 diabetes and obesity, whereby the link to L-cysteine might be relevant in type 2 diabetes development and the link to the fatty acid biosynthesis pathway for body weight gain in response to a carbohydrate-rich diet in obesity development.

## Introduction

Scientific evidence indicates that obesity and obesity-related comorbidities such as type 2 diabetes and cardiovascular diseases are associated with a dysbiosis of the gut microbiome.^1^ Several studies of the last decade imply that environmental factors influence the composition of the gut microbiome^2^ and that the gut microbiome plays a crucial role in the regulation of host metabolism via specific host-microbiome interactions.^1^

Mechanistically, it is thought that a significant elevation in host metabolism-related microbial communities might be associated with an increased capacity to harvest energy from the diet.^3^ In addition, it is now known that an obesity-associated microbiome can alter multiple host factors including inflammation, intestinal permeability, hormonal regulation, bile acids, and lipid metabolism. In the past, nutritional components were thought to be predominant factors inducing alterations of the immune system in obesity (e.g. unsaturated fatty acids^4^), while current evidence highlights the gut microbiome as a key mediator of metabolic inflammation.^5^

Several studies investigated the relation of gut microbial composition at the phylum level to metabolic parameters and dietary components.^6,7^ Recently, however, several pilot projects have targeted specific microbial species, like *Akkermansia muciniphila*, which have been shown to be independently associated with beneficial health traits in obese subjects.^8–10^

In the present study, we followed this more specific approach by focusing on *Parasutterella sp*. at the genus level (and *Parasutterella excrementihominis* at the species level) which belong to the family of *Sutterellaceae*, the order of Burkholderiales, the class of Betaproteobacteria, and the phylum of Proteobacteria.^11^ This microbe is linked to metabolic abnormalities in rodents^12^ and responds to a high fat diet (HFD) intervention in obesity-prone mice.^13^ In addition, recent evidence suggests *Parasutterella* is involved in the mediation of ω3-fatty acid effects on host physiology.^14^ From a mechanistic point of view, *Parasutterella* is associated with both, inflammatory reactions in the intestinal mucosa^15^ and systemic metabolic abnormalities^12^ which might suggest a role in the development of systemic low-grade metabolic inflammation due to dysbiosis. In fact, in a recent MRI (magnetic resonance imaging) study by our group, we found *Parasutterella* associated with hypothalamic inflammation in obese humans^6^ which is thought to interfere with appetite- and satiety regulation contributing to the development of obesity.

Since most published data are on rodent models, the aim of this present, explorative study was to characterize *Parasutterella* more deeply with respect to normal human physiology as well as human metabolic and chronic inflammatory diseases and nutrient intake in order to understand the development of obesity and to identify novel therapeutic approaches for future treatments.^16^ For this purpose, we analyzed 16s amplicon microbiome data in 1,544 subjects of our large FoCus cohort in Kiel, Germany.^17^ The subjects were extensively geno- and phenotyped. In addition, HPLC metabolomics and detailed dietary phenotyping were performed (EPIC protocol^18^) and analyzed with regard to *Parasutterella* in the form of metabolic pathway over-representation analysis and microbial metabolic networks. In a previous analysis using the FoCus cohort, we found the overall gut microbiota composition to be associated with single nucleotide polymorphisms (SNPs) in the human vitamin D receptor (*VDR*) gene locus and the Proopiomelanocortin (*POMC*) gene locus. Therefore, we aimed to analyze as well if *Parasutterella* abundance is determined by these two human genetic factors.

To increase reliability and interpretability, we also performed a human intervention study in order to show that *Parasutterella* is not only associated with an obesity phenotype but also responds to a weight loss therapy indicating a potential functional relevance. In addition, in the analysis of the intervention study, we aimed to quantify *Parasutterella* abundance levels by qPCR not to rely solely on 16S rRNA data.

## Research design and methods

### Study cohorts and study design

#### Cross-sectional cohort (FoCus cohort)

The present investigation included n = 1,544 subjects (63% females) of the northern German Food Chain Plus (FoCus) cohort (established from 2011 to 2015), which has been previously reported.^19^ Subjects were stratified into five groups according to BMI and the presence of type 2 diabetes (Table 1). 422 subjects were enrolled from the obesity outpatient clinic of the Department of Internal Medicine I of Kiel University. The remaining 1,122 subjects were recruited from regional registration offices as cross-sectional controls. Fasted serum and -stool samples as well as anthropometric values were collected at the study center. In addition to these measurements, subjects completed a 12-month retrospective food frequency questionnaire used by the European Prospective Investigation into Cancer Nutrition (EPIC) study. In addition, for validation purpose in 10% of the study probands, an additional 24-h nutrition profile was obtained by a phone interview. The median age of the whole cohort was 52 ± 14.2 years. The study population was generally overweight in Body Mass Index (BMI 27.8 kg/m^2^), with median height being 1.72 m and the median weight being 85 kg. Metabolic parameters including HOMA-IR index indicated low level of insulin resistance (2.42). The fasting glucose level was 95 mg/dL. Serum triglyceride levels appeared to be within a normal range of 108 mg/dL. Inflammatory parameters such as Interleukin-6 (IL-6) (3.7 pg/mL) and C-reactive protein (CRP) (3.3 mg/L) were within the normal range in the study population. This FoCus subset had prevalence for diabetes of 14.3%, whereas 11.9% of the whole subset was affected by type 2 diabetes.

**Table 1.** Descriptive statistics of the three cohorts included in the study showing variables such as age, sex, and BMI. Further division of the cohorts was based on five groups: underweighted, normal weighted, overweight, obese with T2D, and obese without T2D

	*FoCus cohort* *(n = 1,544)*	*ATP cohort* *(n = 438)*	*Intervention cohort* *(n = 55)*
Parameter	Median (25^th^ and 75^th^ percentile) or Mean ±	Median (25^th^ and 75^th^ percentile) or Mean ±	Median (25^th^ and 75^th^ percentile) or Mean ±
Age (years)	51.62 ± 14.22	56.9 ± 6.27	45.79 ± 10.89
Sex (% female)	63	72	69
BMI (kg/m^2^)	27.8 (23.68; 35.9)	30.62 (26.55; 36.88)	45.25 (43.36; 48.13)
Underweighted (<20 kg/m^2^) (%)	4.5	-	-
Normal weighted (20–25 kg/m^2^) (%)	28.6	2.9	-
Overweighted (25–30 kg/m^2^) (%)	27	46	-
Obese (>30 kg/m^2^) with type 2 diabetes (%)	10.6	5.2	17.3
Obese (>30 kg/m^2^) without type 2 diabetes (%)	29.2	45.8	82.7

The study was conducted according to the guidelines laid down in the Declaration of Helsinki and was approved by the ethic committee of the Medical Faculty of the University of Kiel (Germany). All subjects gave their informed consent for study participation and data usage.

#### Validation cohort

To validate the FoCus results, a study population of n = 438 subjects from Alberta’s Tomorrow Project (ATP), a cross-sectional cohort from Alberta Canada was included for comparison^20^ (Table 1). These included 242 healthy controls (with a BMI between 20 and 25 kg/m^2^), 120 subjects with cardiovascular disease, 36 with chronic metabolic disease, and 44 with chronic inflammatory disease. Fecal samples from all n = 438 ATP subjects were available for the present analysis and 16S rRNA gene sequencing was performed in the same laboratory and using the same methodology as for the FoCus samples.

#### Intervention cohort

For validation of 16S rRNA gene sequencing data, we performed quantitative polymerase chain reaction (qPCR) for *Parasutterella excrementihominis* of n = 55 patients of an intervention cohort described elsewhere^21^ (Table 1). In brief, this group of severely overweight patients underwent a dietary intervention that consisted of a very low-calorie formula diet for the duration of 12 weeks, followed by a stabilization period of another 14 weeks. It is important to mention that the formula diet used was enriched in fish oil according to the EU regulations.

Patients received extensive medical, dietary, and psychological monitoring during the intervention and gave blood and stool samples at the beginning, mid-point, and end of the study. All patients of the intervention study gave their informed consent.

### 16S rRNA gene sequencing of the FoCus cohort

Stool samples of the study populations were immediately stored at −80°C and were passed forward to the Institute of Clinical Molecular Biology (IKMB) in Kiel (Germany) for microbiome sequencing. 16S rRNA gene sequencing was performed as explained previously.^22^ Bioinformatic analysis was based on amplicon sequence variants (ASVs) in R. For the FoCus cohort, the median sequencing depth was 36,048 reads, with an IQR from 23,444 to 52,786 reads. The sequencing depth of the Canadian ATP cohort was slightly lower with a median of 22,931 and an IQR of 18,301–28,758. Samples of the FoCus and ATP cohorts were sequenced in the same lab, on the same platform. Samples with a sequencing depth below 10,000 were removed as part of the quality control offered by the sequencing lab. Complete sets of genetic, food questionnaire (EPIC), and microbiome data were available for n = 1,443 individuals.

### Metabolomics sample preparation in the FoCus cohort

Serum samples were thawed on ice and extracted by a modified SIMPLEX approach according to Matyash et al.^23^ From 100 µL blood samples, a lipophilic methyl-tert-butyl ether (MTBE) phase, a hydrophilic methanol-water phase, and a protein pellet were obtained; dried under vacuum (speed-vac from Thermo Fisher, Germany); and resuspended with the following solvent: the lipophilic phase with a mixture of isopropanol/chloroform (3/1, v/v) with 0.1% acetic acid; the hydrophilic phase with water/methanol (50/50, v/v) with 0.1% acetic acid.

To each sample, 4 µL of an internal standard mixture was added. The hydrophilic standard contains a mixture of ^13^C-labeled tyrosine and tryptophan. The lipophilic standard contains synthetic lipids PC 5:0, PC 11:0, PC 19:0, and PG 17:0. All samples were stored at −80°C until the day of measurements.

Mass spectrometry was conducted using a FT-ICR-MS (7 Tesla, SolariXR, Bruker, Bremen, Germany) in the flow-injection mode. The injection was facilitated by a HPLC autosampler (1260 Infinity, Agilent, Waldbronn, Germany). The eluent for the hydrophilic samples was water/methanol (50/50, v/v) with 0.1% acetic acid and for the lipophilic samples, isopropanol/chloroform (3/1, v/v) with 0.1% acetic acid, respectively. The samples were ionized with an electrospray ionization source (in both modes). Different methods were used, each optimized to the respective detection range (in total, the range was from 65 to 3000 m/z). The average resolution at 400 m/z was 600,000. Evaluation of mass features was conducted with DataAnalysis 5.0 and MetaboScape 4.0.1 both from Bruker (Bremen, Germany). Sum formulas were calculated based on the mass error and isotopic fine structure of mass features. To reduce false-positive results, the seven golden rules of Kind and Fiehn were applied.^24^

### Metabolomics pathway analysis

Based on the results of mass spectrometry metabolite analysis, we performed an enrichment analysis in MetaboAnalyst 5.0 in order to find common pathways of all the metabolites that were significantly associated with *Parasutterella*. Out of 256 nominally significant metabolites, we made use of n = 126 metabolites that were significantly associated with Parasutterella abundance after FDR correction of p-values in the count part of the Hurdle model. Of the 126, a total of 76 metabolites could be matched in terms of The Small Metabolites Pathway Database (SMPDB), whereas the other 40 were unidentified compounds.

### Microbial community metabolism

We used metabolic network modeling to elucidate to which extent *Parasutterella* and associated gut microbes are involved in the consumption or production of certain metabolites, like L-cysteine. In order to predict L-cysteine consumption by bacteria of the gut microbiota, we employed gapseq (v1.2) to reconstruct metabolic models^25^ based on genomic data originating from the reference set of 820 bacteria and archaea belonging to the human gut microbiota (AGORA collection)^.(25)^ Default settings of gapseq were used. For gap filling and modeling of in silico growth, we assumed a nutritional environment corresponding to the average dietary input recorded for a cohort of human participants as described previously (“Kiel cohort”).^26^ For each individual bacterial model, we used flux balance analysis^27^ implemented in the R-package Sybil version 2.2.0^28^ with biomass production as objective and unconstrained L-cysteine uptake as input to predict maximal L-cysteine consumption during optimal growth. We used information about the relative abundances of bacterial species from the FoCus cohort as references to determine the average abundance of each bacterial species across human participants. Relative cysteine uptake was then determined by multiplying the maximal L-cysteine uptake of each bacterial species by the average relative abundance of the corresponding bacterial species in the FoCus cohort.

### Quantitative polymerase chain reaction

Genomic DNA (gDNA) was extracted from stool samples using the QIA amp Fast DNA Stool Mini Kit according to the manufacturer’s protocol. We decided for measuring *Parasutterella excrementihominis* since the DNA for the genus of *Parasutterella* was not available at DSMZ, Germany. Furthermore, we found appropriate primers for *Parasutterella excrementihominis* in the literature. This species is known to be the primary species of intestinal *Parasutterella* at least in our 16S rRNA sequencing data of the FoCus cohort (shotgun analysis). For quantifying the amount of DNA in each fecal sample, we used a Thermo NanoDrop 2000 spectrophotometer. A concentration of 2.5 ng/µL was used of each sample. For qPCR, a tenfold serial dilution of *Parasutterella excrementihominis* was generated to produce a standard curve (ordered from DSMZ, Germany). The qPCR system contained a PowerUp^TM^ SYBR^TM^ Green Master Mix (5 µL), the forward primer and reverse primer (2.5 µM each), and DNAse free water (4.5 µL). The primers were selected according to a study of Chen and coworkers: GGAAGTACGGTCGCAAGA (forward) and TGTCAAGGGTTGGGTAAGACA (reverse).^29^ Melt curves and relative quantification of *Parasutterella excrementihominis* were generated with Bio-Rad CFX Connect^TM^ Real-Time System PCR instrument with the help of the following template: 50°C for 2 min, preliminary denaturation at 95°C for 2 min, 40 cycles at 95°C (15 s), 40 cycles annealing at 60°C (30 s), and 40 cycles extension at 60°C (1 min). Melt curve analysis was performed between 65°C and 95°C (increment: 0.5).

Bio-Rad CFX Manager 3.0 was used to analyze melt curves and copy numbers of the target gene concentrations. C_t_-values of the target gene and the standard curve indicated the final concentration through the following formula: y = −1.48lg(x) + 13.854 (R^2^ = 0.9979).

### Genotyping

FoCus probands were genotyped using the Iscan Immunochip Opticall and the Iscan Omniexpress Exome Chip. Quality control and preprocessing of genetic data are described in detail elsewhere.^30^ For the purpose of this study, the *VDR* and *POMC* genes were defined as genes of interest. Quality control of genetic data was done in PLINK v. 1.9.

57 SNPs in the *VDR* locus (chr 12, 48.22–48.32 Mb) and 26 SNPs in the *POMC* locus (chr2, 25.36–25.46Mb) passed quality control and filtering.

Kruskal-Wallis Test was used in R to test for associations between SNP genotypes and *Parasutterella sp.* abundance groups.

### Statistical analysis

Statistical significance was set at *P* < .05 P-values are shown as FDR (False Discovery Rate) adjusted to correct for multiple hypotheses testing.

#### Univariate analysis

The statistical and graphical data analysis was done in Rv3.6.0. Data were tested for normality by Shapiro-Wilk-Test and are presented as means ± SDs (standard deviation) for normally distributed variables and as median for non-normally distributed variables. In the intervention study, paired Wilcoxon signed-rank tests (for non-normally distributed variables) have been used for metric variables to determine differences between two time points in one group.

#### Multivariate analysis

Since *Parasutterella* abundances in the explorative part of this study were derived from 16s rRNA amplicon sequence data, Hurdle models were chosen in order to handle the excess number of zeros and overdispersion in the data. Hurdle models are two-part (negative) binomial regression models in which probabilities for the non-zero and zero abundances of *Parasutterella* are handled separately. In the first part of the model (henceforth called “count part”), the model is truncated at zero and a negative-binomial linear regression is fitted to the remaining abundance data. In the second part of the model (henceforth called “zero part”), a logistic regression is fitted to determine the binary probability of *Parasutterella* abundance being zero vs. non-zero.

ASV abundances of *Parasutterella sp.* lower than 10 counts were set to zero before using the Hurdle algorithm to adjust for the error in determining low counts of 16S rRNA data. A multivariate Hurdle algorithm was applied to model the association of *Parasutterella* and the independent variable of interest for dietary data, phenotypes linked to obesity, and metabolic health and clinical biomarkers. Since inflammatory comorbidities (inflammatory bowel disease (IBD) and psoriasis) other than metabolic diseases that are known to affect the microbial composition, the presence of such diseases and the proband age were included as covariates.^31,32^ In some analysis when marked accordingly, we additionally used BMI as a covariate.

Metabolomics data was either K-nearest neighbor (KNN) (for samples with < 50% missingness) or limit of detection (LOD) (for samples with > 50% missingness) imputed and then log-transformed. For the metabolomics analysis, once again the Hurdle models were used as previously described, since the resulting data matrix consists of zero-inflated integers, which can statistically be treated similarly to gene-based count data. For the peak annotation, a customized and local database was established, which contained sum formula and names of all metabolites of interest. The database was used for annotation with MetaboScape and the resulting data frame contained information about each mass error and isotopic fine structure (as the mSigma value). By means of these both values, the resulting metabolites were validated (only Metabolites with an error below 2 ppm and a “good” mSigma (< 200) were accepted for further statistical evaluation).

For microbiomics analysis, subjects were grouped into high (>10) or low (≤10) counts of *Parasutterella* and Mann-Whitney-U test were used to find differences between the two groups. Microbiome β- and α- diversity was characterized by the Bray-Curtis dissimilarity index and the Shannon, Chao1, and Species Richness indices, respectively, and compared between the groups. Differential abundance between BMI groups was tested with the DESEQ2 integration for phyloseq data in R. To evaluate classifier performance in human obesity of *Parasutterella* in relation to *Akkermansia*, prediction models were built using random forests and ROC curves and AUC values were determined. For some of these analyses, the FoCus cohort was stratified into five groups concerning BMI and diabetes status (see Table 1).

## Results

To generate a comprehensive overview of *Parasutterella* on the development of obesity and type 2 diabetes, we decided to investigate the bacterium on metabolic, inflammatory, dietary, microbiome, metabolome, and genetic level. Additionally, we examined qPCR-determined *Parasutterella* abundances in a weight loss intervention study to further validate bioinformatical calculations and to gain insight into the functional capabilities of this species.

As described in the method section, we used two-part Hurdle models to test for association between our independent variables of interest and *Parasutterella* abundance. Since we did not observe any significant correlation in the zero part of each model (logistic regression) except of microbiomics calculations, the results described in the following pages refer solely to the count part of the Hurdle models.

### *Parasutterella* and measures of obesity

**Figure 1.**
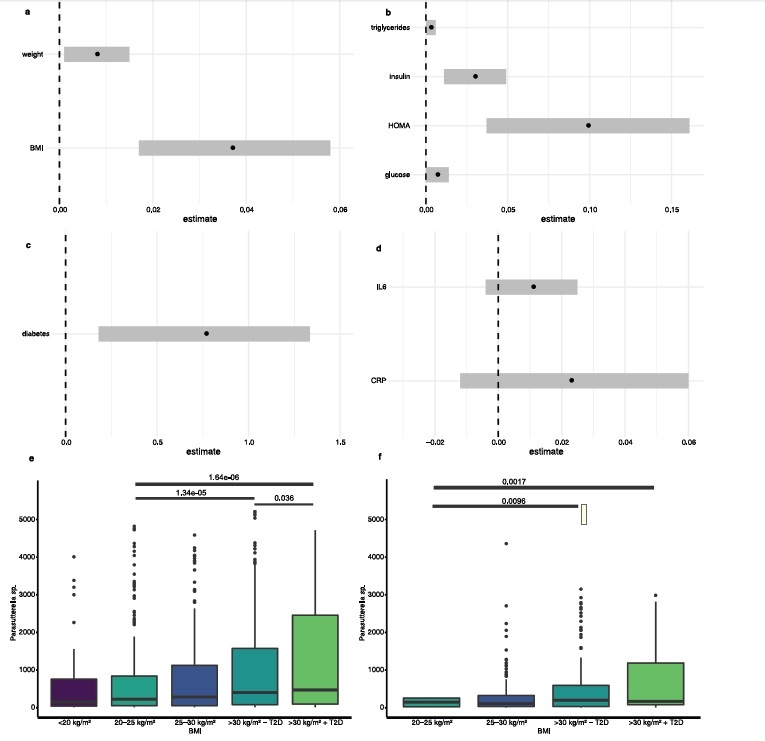
*Parasutterella and obesity, glucose and lipid abnormalities as well as metabolic inflammation*. (a) Association of *Parasutterella sp*. with weight and BMI in n = 1,544 subjects reported through estimate and standard error (Hurdle count model). (b) Association of *Parasutterella sp.* with metabolic parameter in n = 1,544 subjects reported through estimate and standard error (Hurdle count model). (c) Association of *Parasutterella sp.* with diabetes in n = 1,544 subjects reported through estimate and standard error (Hurdle count model). (d) Association of *Parasutterella sp.* with inflammatory parameters in n = 1,544 subjects reported through estimate and standard error (Hurdle count model). (e) *Parasutterella sp.* abundance in relation to BMI and T2D groups in the FoCus cohort. (f) *Parasutterella sp.* abundance in relation to BMI and T2D groups in the ATP cohort. While the sequencing depth of the ATP cohort was slightly lower overall than in FoCus (median FoCus: 36,048; median ATP: 22,931), Figures 1e and f demonstrate that the distribution of *Parasutterella* abundance in relation to BMI is comparable in both cohorts and that *Parasutterella* is a highly abundant microbe in ATP as well, although the height of box plots differs slightly between cohorts.

We first examined 1,544 subjects of the FoCus cohort regarding the obesity phenotype. Therefore, we used *Parasutterella sp.* > 10 counts (n = 1132 samples that met the threshold of *Parasutterella sp*. > 10 counts) for multivariate modeling in regard to different metric variables such as BMI and body weight. This threshold was recommended by QIIME pipeline (https://docs.qiime2.org/2021.4/) in order to only incorporate values of *Parasutterella sp.* that were truly measured through 16S rRNA gene sequencing. We found a significant positive correlation between positive counts of *Parasutterella sp.* abundance and BMI (3.71e^−2^, *P* = 3.11e^−3^) (Figure 1a), meaning that there was an increased *Parasutterella sp.* abundance in subjects with higher BMI. The results were in line with data regarding weight measurements: there was a nominally significantly positive association between *Parasutterella sp.* abundance and weight of the subjects (7.95e^−3^, *P* = 2.0e^−2^).

The significant positive association of *Parasutterella* and BMI found in the European FoCus cohort was validated in the independent Canadian ATP cohort (n = 305 samples that met the threshold of *Parasutterella sp*. > 10 counts). Of interest, we found that *Parasutterella sp.* abundance was increased in probands with higher BMI (*P* = 2.0e^−3^) in the Canadian cohort as well.

### *Parasutterella* and diabetes phenotypes

In the next step, we analyzed glucose metabolism and found *Parasutterella sp.* abundance to be positively associated with elevated HOMA (9.88e^−2^, *P* = 1.5e^−2^) and fasting insulin (3.02e^−2^, *P* = 1.38e^−2^) (Figure 1b). In addition, we found a positive association between *Parasutterella sp.* abundance and fasting glucose (6.81e^−3^, *P* = 5.38e^−2^). Furthermore, there was a significantly positive association between *Parasutterella sp.* abundance and the presence of diabetes type 2 (7.58e^−1^, nominal significant, *P* = 1.02e^−2^) (Figure 1c). With regard to lipid metabolism, *Parasutterella sp.* was nominally positively associated with serum triglyceride levels (2.61e^−3^, *P* = 3.34e^−2^) (Figure 1b). However, when adapting for BMI, the significant association to the measures of insulin sensitivity diminished, suggesting the association of *Parasutterella* with insulin resistance to be indirectly, mediated via its effect on body weight gain.

In a subsequent analysis, we stratified the FoCus cohort into five groups concerning BMI and diabetes status (see Table 1) and found a significant difference between the lean and the obese group concerning *Parasutterella* abundance (Figure 1e)). Of interest, a significant difference in *Parasutterella* abundance was further identified between obese probands without type 2 diabetes compared to obese probands with type 2 diabetes, whereby diabetics had around 70% higher *Parasutterella* abundance (Figure 1e). In the ATP cohort, a similar pattern was observed, whereby the difference between obese individuals with and without type 2 diabetes did not reach statistical significance, which might be due to the smaller number of subjects in ATP (n = 438, 5.2% diabetics) compared to FoCus (n = 1,544, 10.6% diabetics) (Figure 1f).

### *Parasutterella* and inflammatory phenotypes

In order to gain insights into the inflammatory properties of *Parasutterella sp*., we examined two biomarkers that mirror the inflammatory state of the subjects. *Parasutterella* sp. abundance was neither associated with CRP nor with IL-6 (2.39e^−2^, *P* = 1.9e^−1^; 1.07e^−2^, *P* = 1.5e^−1^) (Figure 1d). This finding indicates no major association of *Parasutterella sp*. to systemic metabolic inflammation.

### *Parasutterella* and dietary phenotypes

Dietary data were derived from 12-month food frequency questionnaires (n = 1,443). Analysis of 19 dietary components revealed that the intake of total carbohydrates showed a nominal significant positive correlation with *Parasutterella sp*. abundance (4.51e^−2^, *P* = 4.24e^−3^) (Table 2) falling in line with the data on diabetes phenotypes. The composition of total carbohydrate intake consisted of 22.09% monosaccharides, 31.56% disaccharides, 44.22% polysaccharides, and minor parts of sugar alcohol and oligosaccharides. Appropriately, monosaccharides showed a nominal significant positive association with *Parasutterella sp.* abundance (1.115e^−2^P = 1.19e^−3^). In contrast, the total fat intake of the subjects was nominally significantly negatively associated with *Parasutterella sp.* abundance (−5.13e^−2^, *P* = 5.97e^−3^). In more detail, we found the polyunsaturated fatty acid (PUFA) linolenic acid (ω3-fatty acid) nominally significantly negatively associated with *Parasutterella sp.* abundance (−3.3e^−1^, *P* = 4.99e^−2^). Furthermore, eicosenoic acid was nominally significantly negatively associated with *Parasutterella sp.* (−3.25, *P* = 3.04e^−3^). The mean percentage of the intake of total fiber was 22.3%. Dietary data were additionally adjusted for BMI.

**Table 2.** Dietary parameters regarding the abundance of *Parasutterella sp.* (two-part Hurdle model), truncated linear model considering only counts of *Parasutterella sp.* (count part). Dependencies of parameters and the abundance of *Parasutterella sp.* reported through estimate, confidence intervals, and p-values in the respective model. First part of the Hurdle model considers only counts of *Parasutterella sp.* using a negative binomial regression. After FDR-correction, p-values were not significant

Parameter	Estimate	Confidence interval [2.5%, 97.5%]	p-value
Carbohydrates (g/day)	4.51e^−2^	[1.42e^−2^, 7.60e^−2^]	4.24e^−3^
Monosaccharides (g/day)	1.15e^−2^	[4.53e^−3^, 1.88e^−2^]	1.19e^−3^
Protein (g/day)	3.76e^−2^	[8.03e^−2^, 1.56e^−1^]	5.31e^−1^
Fat (g/day)	−5.13e^−2^	[−8.78e^−2^, 1.47e^−2^]	5.97e^−3^
Linolenic acid (g/day)	−3.3e^−1^	[−6.61e^−1^, −2.63e^−5^]	4.99e^−2^
Eicosenoic acid (g/day)	−3.25	[−6.21, −3.07e^−1^]	3.04e^−2^
Butanoic acid (g/day)	3.07e^−1^	[−7.99e^−1^, 1.84e^−1^]	2.2e^−1^
Hexanoic acid (g/day)	−4.84e^−1^	[−1.25, 2.85e^−1^]	2.17e^−1^
Vitamin D (mg/day)	−72.75	[−1.58e^+2^, 13.13]	9.68e^−2^
Vitamin B9 (mg/day)	2.61	[−6,24, 11.46]	5.62e^−1^
Vitamin B6 (mg/day)	−7.51e^−1^	[−1.48, −2.14e^−2^]	4.36e^−2^
Vitamin C (mg/day)	2.04e^−3^	[−2.14e^−3^, 6.21e^−3^]	3.39e^−1^
Vitamin B12 (mg/day)	−45.81	[−1.75e^+2^, 83.74]	4.88e^−1^
Vitamin E (mg/day)	−3.65e^−2^	[−1.01e^−1^, 2.8e^−2^]	2.68e^−1^
Iodine (mg/day)	4.16	[−1.37, 9.7]	1.41e^−1^
Iron (mg/day)	2.28e^−1^	[7.99e^−2^, 3.77e^−1^]	2.55e^−3^
Calcium (g/day)	3.55e^−1^	[−7.35e^−1^, 1.44]	5.23e^−1^
Magnesium (g/day)	−5.35e^−1^	[−4.35, 3.28]	7.83e^−1^
Zinc (mg/day)	−3.98e^−2^	[−1.52e^−1^, 7.21e^−2^]	4.85e^−1^

Besides macronutrients, we examined several micronutrients, whereby iron was nominally significantly positively associated with *Parasutterella sp*. abundance (2.28e^−1^, *P* = 2.55e^−3^). Regarding vitamins, a nominal significant negative association was found for vitamin B6 and *Parasutterella sp.* (−7.51e^−1^, *P* = 4.36e^−2^).

Having found an association of *Parasutterella sp*. abundance with carbohydrate intake we also examined carbohydrate intake independently of the *Parasutterella* abundance and found a significant higher intake in obese individuals compared to lean controls (*P* = 3.2e^−2^). Importantly, the association of *Parasutterella sp*. and the carbohydrate intake remained significant when adjusting for BMI.

### *Parasutterella* and microbiomics

**Figure 2.**
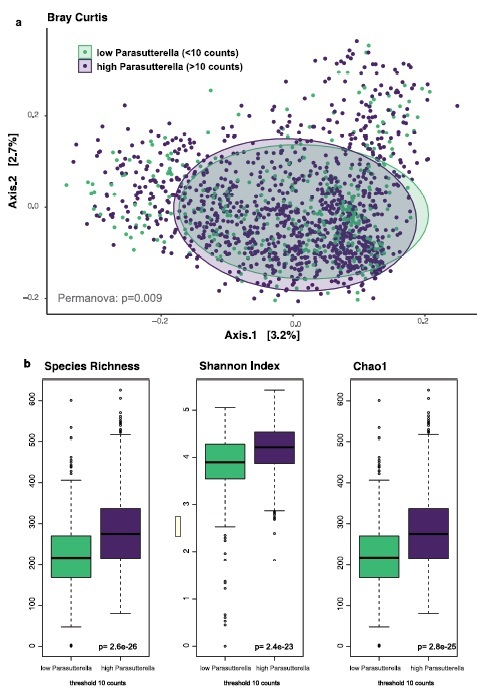
*Parasutterella* and gut microbiome diversity measures. Beta diversity was assessed by Bray-Curtis distance and PERMANOVA. Alpha diversity was assessed by species richness, Chao1 Index and Shannon Index. Statistical significance between high and low Parasutterella groups was tested by Wilcoxon tests.

A comparison of *Parasutterella sp*. levels with the general microbiome composition revealed significant differences in β-diversity measures (Bray-Curtis distance: *P* = 9.0e^−3^, Jaccard index: *P* = 8.0e^−4^) between groups with low versus high *Parasutterella sp*. abundance (Figure 2a). Likewise, α-diversity was significantly reduced in individuals with low *Parasutterella sp*. abundance (zero part of the Hurdle model: Shannon index: *P* = 2.4e^−23^, Chao Index: *P* = 2.8e^−25^, Species richness: *P* = 2.6e^−26^) (Figure 2b). In the count part of the Hurdle model, no significant correlation with *Parasutterella sp*. abundances was detected.

**Figure 3.**
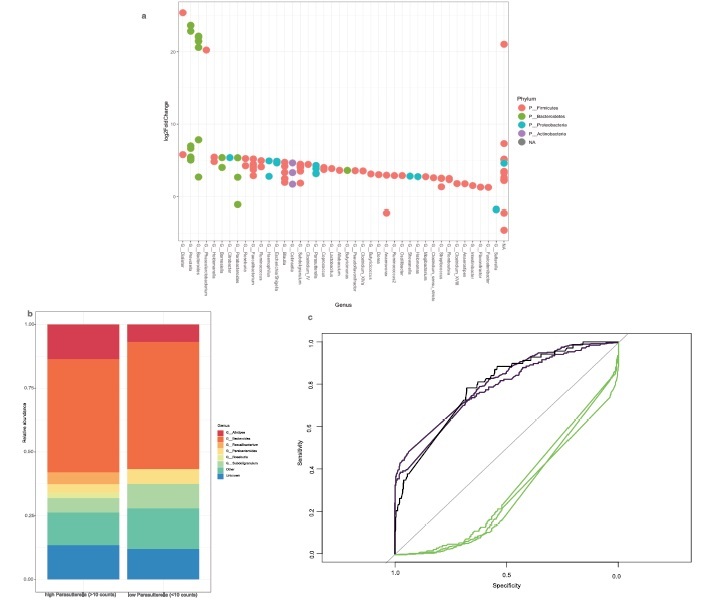
*Parasutterella in relation to other gut microbiome species in human obesity*. (a) LogFold Changes of differentially abundant microbes in human obesity in the FoCus cohort. Comparison was made between the normal weight group (BMI < 25) and the obese group (BMI > 30, without T2D). *Parasutterella sp*. is among the top 20 (=3%) differentially abundant species out of 665 species tested. Plot shows the top 50 differentially expressed species, eight species could not be assigned to a genus (marked NA in the plot). *Parasutterella sp*. is placed 18th among the top 50. (b) Composition plots of probands with high and low (threshold <10 counts) *Parasutterella sp*. (c) ROC curves for prediction models (random forests) of BMI and T2D groups by *Akkermansia* (green, AUC: 0.22–0.28) and *Parasutterella* (purple, AUC: 0.83–0.87) abundance. Comparisons were made between the normal weight group and the (1) obese group without diabetes, (2) obese group with diabetes, and (3) the overweight group according to Table 1. Classifier performance was tested using bootstrapping of AUC results and revealed significantly better performance of *Parasutterella* compared to *Akkermansia* in all comparison groups (*P* = 5.2e-191;-2.8e-185).

The association of obesity and the gut microbiome was evaluated by differential Fold Change analysis. In this analysis, *Parasutterella* was among the top 3% differentially abundant species (placed 18^th^ out of 665 species tested) and consistently exhibited higher fold changes than *Akkermansia sp.*, a species that is an already established marker for obesity (Figure 3a). We have also performed a differential abundance analysis on the genus level (Figure 3b), which revealed that subjects with high *Parasutterella* abundance are characterized by nominally higher abundances of *Alistepes* (r2 = 0.43, *P* = .02), *Faecalibacterium* (r2 = 0.58, *P* = .01), and *Roseburia* (r2 = 0.47, P =  .02). Probands with low counts of *Parasutterella* are in turn characterized by higher abundances of *Bacteroides* and *Subdoligranulum*.

Finally, an ROC analysis revealed that in the FoCus cohort, increased abundance of *Parasutterella* is an even better classifier for obesity compared to a reduced abundance of *Akkermansia*. (Figure 3c). AUCs of *Parasutterella* ranged from 0.83 to 0.87, while *Akkermansia* had AUCs ranging from 0.22–0.28. Differences in AUC for *Parasutterella* and *Akkermansia* were significant for each comparison between normal weight, obese, and diabetes groups (*P*_*NW*_:OBS-T2D = 5.2e-191; *P*_*NW*_:OBS+T2D = 1.4e-171; *P*_*NW*_:Overweught = 2.8e-185, according to the BMI groups shown in Table 1).

### *Parasutterella* and host metabolomics

Presently, MS-metabolomics analysis data are available of n = 470 subjects of the FoCus cohort, consisting of n = 178 metabolically diseased subjects and 292 healthy controls. This analysis took n = 955 detectable metabolites into account. 269 metabolites were nominally associated with *Parasutterella* abundance, of which 126 remained significant after BH adjustment for multiple comparisons. Of interest, we identified L-cysteine (LC) as a metabolite that showed a significant inverse correlation with *Parasutterella sp*. abundance in the count part of the Hurdle model (−13.00, *P* = 1.78e^−3^) (Table 3). Furthermore, 6-Hydroxynicotinic acid was positively correlated with *Parasutterella sp*. abundance (1.57, *P* = 1.22e^−2^) (Table 4). Tables 3 and 4 show the five highest estimates either negative or positive out of in total n = 126 significant metabolites in association with *Parasutterella sp*. abundance in the count part. There were no significant associations in the zero part of the Hurdle model for all metabolites analyzed.

**Table 3.** Metabolomic parameters regarding the abundance of *Parasutterella sp*. (two-part Hurdle model) showing negative associations by using a truncated linear model considering only counts of *Parasutterella sp*. (count part). Dependencies of parameters and the abundance of *Parasutterella sp*. reported through estimate, confidence intervals, and FDR- adjusted p-values in the respective model. First part of the Hurdle model considers only counts of *Parasutterella sp*. using a negative binomial regression. Parameters are chosen considering the five highest estimates

Parameter	Estimate	Confidence interval [2.5%, 97.5%]	p-adjusted
L-Cysteine	−13.00	[−18.89, −7.11]	1.78e^−3^
19,20-DiHDPA	−6.74	[−10.45, −3.02]	1.11e^−2^
Hydroxycholesterol	−4.60	[−7.43, −1.78]	1.98e^−2^
Tetrahydrocortisone	−3.93	[−6.62, −1.24]	3.94e^−2^
Tetracosatetraenoic acid	−3.62	[−5.34, −1.89]	3.04e^−3^

**Table 4.** Metabolomic parameters regarding the abundance of *Parasutterella sp*. (two-part Hurdle model) showing positive associations by using a truncated linear model considering only counts of *Parasutterella sp*. (count part). Dependencies of parameters and the abundance of *Parasutterella sp*. reported through estimate, confidence intervals, and BH- adjusted p-values in the respective model. First part of the Hurdle model considers only counts of *Parasutterella sp*. using a negative binomial regression. Parameters are chosen considering the five highest estimates

Parameter	Estimate	Confidence interval [2.5%, 97.5%]	p-adjusted
[(2 R)-1-[2-aminoethoxy(hydroxy)phosphoryl]oxy-3-[(1Z,11Z)-octadeca-1,11-dienoxy]propan-2-yl] (9Z,12Z,15Z)-octadeca-9,12,15-trienoate	3.53	[1.11, 5.95]	3.99e^−2^
Oxoglutaric acid	1.99	[0.55, 3.44]	5.36e^−2^
6-Hydroxynicotinic acid	1.57	[0.68, 2.46]	1.22e^−2^
Prostaglandin f1 alpha	1.33	[0.80, 1.87]	1.57e^−4^
Isocaproic acid	1.25	[0.61, 1.88]	5.52e^−3^

**Figure 4.**
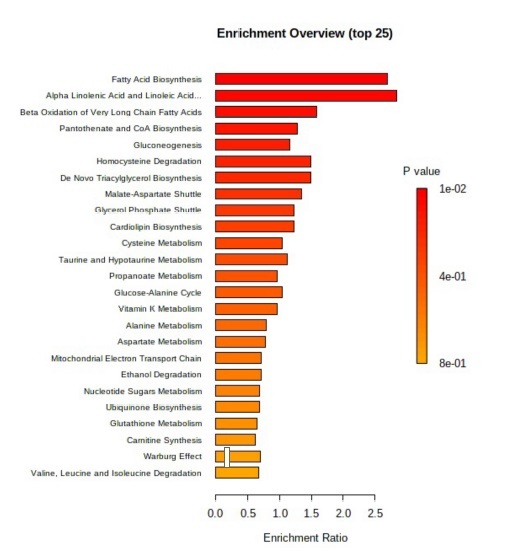
Parasutterella and metabolic pathway enrichment analysis.

Second, the pathway enrichment analysis of 76 metabolites that were successfully matched to terms of the SMPDB database resulted in two nominally significant pathways: fatty acid biosynthesis and α-linolenic acid metabolism (*P* = 1.16e^−2^, *P* = 4.62e^−2^) (Figure 4). All other pathways in the figure were not significantly enriched.

Enrichment ratio and related p-value of the pathway enrichment analysis (SMPDB database) displaying fatty acid biosynthesis and α-linolenic acid metabolism as nominally significant (*P* < .05).

### Microbial metabolic network analysis

We used metabolic network modeling to investigate maximal L-cysteine consumption by member species of the human gut microbiota. This analysis revealed that *Parasutterella* is among the top five consumers, together with *Dialister invisus, Enterobacter cancerogenus, Prevotella copri*, and *Escherichia coli* K12. Hence, the fact that *Parasutterella* is a high L-cysteine consumer fits with the findings of reduced L-cysteine levels in the serum of human subjects with high *Parasutterella* abundance. Table 5 gives an overview of the top L-cysteine consumers in the gut microbiome.

**Table 5.** Gut bacteria on species level with the highest relative L-cysteine consumption in human samples. The second column indicates the maximal predicted L-cysteine consumption of each strain during optimal growth, the third column the average abundance in the FoCus cohort and the fourth column the abundance-weighted cystein consumption. The fifth column shows the relative L-cysteine consumption as a sum of 1 regarding microbiome abundance in the FoCus cohort

Species (strain designation in the AGORA collection)	Maximal L-cysteine consumption (mmol/gDW/h)	Average relative abundance	Abundance-weighted cystein consumption	Relative L-cysteine consumption
*Dialister invisus* DSM 15470	40.8	0.078	3.20	13.72%
*Enterobacter cancerogenus* ATCC 35316	97.7	0.027	2.68	11.53%
*Escherichia coli* K12 MG1655	47.1	0.052	2.47	10.59%
*Prevotella copri* CB7 DSM 18205	38.9	0.058	2.27	9.77%
*Parasutterella excrementihominis* YIT 11859	81.9	0.026	2.12	9.10%
*Sutterella wadsworthensis* 3145B	96.3	0.008	0.81	3.48%
*Roseburia hominis* A2 183	89.4	0.008	0.70	3.02%
*Hafnia alvei* BIDMC 31	98.1	0.007	0.70	3.01%
*Bilophila wadsworthia* 316	96.3	0.005	0.51	2.17%
*Acidaminococcus intestini* RyC MR95	88.2	0.004	0.39	1.68%

### *Parasutterella* and host genomics

In total, we examined 83 different SNPs. None of the SNPs at the *VDR* locus were significantly associated with *Parasutterella* abundance. At the *POMC* locus, wild-type homozygosity of the SNP rs7565877 was nominally significantly associated *(P = *3.0e^−2^) with abundance of *Parasutterella*.

### *Parasutterella* and weight loss intervention

**Figure 5.**
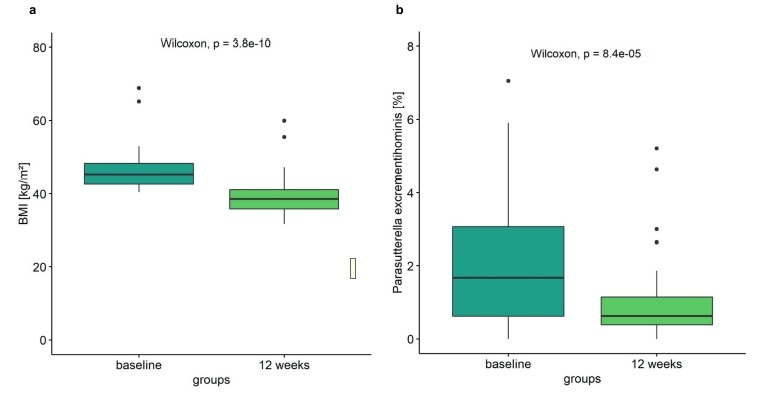
*Parasutterella and human weight loss intervention*. (a) Difference of BMI in subjects of intervention at baseline compared with 12 weeks (Wilcoxon signed-rank test, *P* < .05), (n = 55). (b) Abundance of *Parasutterella excrementihominis* in subjects of intervention at baseline compared with 12 weeks (Wilcoxon signed-rank test, *P* < .05), (n = 55).

The patients of the weight loss intervention lost a significant amount of weight (mean of 23 kg; Wilcoxon paired, *P* = 5.30e^−10^). BMI dropped from 45.2 kg/m^2^ to 38.5 kg/m^2^ (Figure 5a). Of interest, *Parasutterella excrementihominis* was significantly reduced during the intervention (Figure 5b). The median value of relative *Parasutterella excrementihominis* abundance at baseline was 1.62% in comparison to 0.62% of *Parasutterella excrementihominis* after the intervention. These data indicate that *Parasutterella* is not only associated to the obesity phenotype in a steady state but also reacts significantly to variations in body weight. However, it has to be mentioned that the formula diet used within the human intervention study has a low carbohydrate content which also might have affected the change in *Parasuterella* abundance.

### Comparability of cohorts used in this study

We performed the following analysis to ensure that the ethnically and obesity-status diverse cohorts in this study allow for comparable results. Figure 1e and 1f provide an overview of the distribution of *Parasutterella* abundances in relation to BMI for both the FoCus cohort and the Canadian ATP cohort. Furthermore, we compared the dietary patterns in both cohorts by calculating the Mediterranean Diet Score, a score that is commonly used to evaluate healthy dietary patterns. There was no significant difference in adherence to the Mediterranean diet between the German and Canadian cohort (Supp. Figure 1). Lastly, we compared the microbial composition of the population-based FoCus cohort with the intervention cohort at baseline (Supp. Figure 2) and found no significant difference (*P* = .872, multivariate comparison of abundance at phylum level). Overall, we conclude that the cohorts in this study can justifiably be compared to each other.

## Discussion

In recent years, several studies have investigated the relation of gut microbial composition at the phylum level to obesity and its metabolic comorbidities. Due to previous findings in various rodent models, we further characterized a specific bacterium, *Parasutterella*, at the genus level using more than 1.500 subjects from the FoCus cohort in Kiel. We observed a positive association between *Parasutterella* abundance and obesity, as well as type 2 diabetes and carbohydrate-rich food intake. Additionally, *Parasutterella* was significantly reduced after a sustained weight loss intervention in subjects with obesity and associated with L-cysteine among other metabolites. Metabolite pathway analysis revealed an enrichment of the fatty acid biosynthesis pathway.

Our data indicate a positive association between *Parasutterella* and obesity, which we were able to validate in the independent Canadian ATP cohort. These results are in line with research of mechanistic studies in animal models from independent groups. For example, Gu et al. found that *Parasutterella sp*. was significantly enriched in obesity-prone mice in comparison to obesity-resistant mice both fed a HFD.^13^ Furthermore, bacteria of Proteobacteria phylum, to which Parasutterella *sp*. belongs, are known to be highly abundant in obesity and other metabolic diseases.^33,34^ In addition, several authors classify Parasutterella *sp*. as pro-diabetic in animal studies. For instance, in a study by Cheng et al., the authors reported that mice fed with galacto-oligosaccharides (GOS) exhibit a change in their microbiota with a significant enrichment in *Parasutterella sp*. and at the same time a significantly increased blood glucose level compared to control animals.^35^ This is in line with our finding that *Parasutterella sp*. is elevated in patients with type 2 diabetes.

Dietary patterns are a key factor in determining gut microbiota composition in humans as well as the pathogenesis of obesity.^36^ As such, we examined the role of diet in *Parasutterella sp*. abundance. Therefore, in the present analysis, we made use of the detailed EPIC dietary data available for all FoCus subjects. Our data indicate that carbohydrate (especially monosaccharides) and ω3 linolenic acid intake are associated with the abundance of our target bacterium. A higher intake of carbohydrates was associated with a higher abundance of *Parasutterella sp*., whereas a higher intake of linolenic acid was associated with a lower abundance of *Parasutterella sp*. In a previous study using the FoCus cohort, we found that polyunsaturated fatty acids are positively associated with the β-diversity of microbial composition.^30^ Hence, the negative association of *Parasutterella* with ω3 linolenic acid fits into the concept that this bacterium is related to negative cardiovascular effects.^37^ This theory is further supported by the metabolic pathway enrichment results, which showed that many of the metabolites that are associated with *Parasutterella* are involved in the fatty acids synthesis pathways.

By using our untargeted HPLC-based mass spectrometry metabolomics data set, L-cysteine revealed an inverse correlation with *Parasutterella sp*. displaying a convincing estimate that underlines the fact that L-cysteine is linked to blood glucose control which was described in the literature.^38^ Intriguingly, metabolic network modeling revealed *Parasutterella sp*. as a potential top consumer of L-cysteine within the human gut microbiota. Furthermore, a study of Ju et al. investigated mice colonized with *Parasutterella* which had significantly reduced concentrations of taurine (degradation product of cysteine) and N-Acetyl-DL-methionine that implies *Parasutterella* to be able to reduce also cysteine associated metabolites.^39^

So far, it is known that supplementation of L-cysteine improves glycemia and also lowers vascular inflammation in diabetic rodents^38^ meaning L-cysteine reveals anti-diabetic characteristics. In rats that were fed a high-sucrose diet and a whey concentrate rich in L-cysteine, the data showed that an increase in L-cysteine limited the impairment of glucose homeostasis through lowering oxidative stress.^40^ Of interest, especially pancreatic β cells are extremely susceptible to oxidative stress due to a high endogenous production of reactive oxygen species (ROS) and a low expression of antioxidative enzymes.^41^ Since we found that *Parasutterella* is associated with type 2 diabetes but not with (BMI-independent) measures of insulin resistance, this finding might point toward abnormalities in insulin secretion due to *Parasutterella* promoting type 2 diabetes development.

In a second analysis, we examined the enrichment of metabolic sets and found an association of *Parasutterella sp*. abundance with the fatty acid biosynthesis pathway. This finding falls in line with the fact that elevated BMI and body weight linked to an obese phenotype have elevated fatty acid biosynthesis systemically. Of interest, it is known that *Parasutterella secunda* does not produce significant amounts of propionate^11^ and *Parasutterella excrementihominis* is producing only traces of propionate.^42^ Since SCFA are known to inhibit human fatty acid biosynthesis and induce human fatty acid oxidation,^43^ this might suggest a mechanism on how *Parasutterella* induces human fatty acid biosynthesis as found in our analysis. However, we have to admit that (1) we did not measure SCFA fecal concentrations in the present analysis in relation to *Parasutterella* abundance and (2) that in our differential abundance analysis *Parasutterella* is correlated with bacteria that are SCFA producers, e. g. *Faecalibacterium* and *Roseburia*. Hence, while our human cohort study can only identify association, the data presented here might serve as a basis for future intervention studies in model systems of obesity and type 2 diabetes to identify the exact molecular and cellular mechanisms on how *Parasutterella* affects fatty acid biosynthesis.

Due to our previous findings on SNPs in the human *VDR* influencing gut microbiome composition, we decided to include human genetic factors in our present analysis. However, none of the SNPs at the *VDR* locus were significantly associated with *Parasutterella sp*. abundance. Furthermore, we examined common SNPs in the *POMC* locus, since these were also found to influence the gut microbiome in our previous study^30^ and SNPs in the *POMC* gene are associated with body weight dysregulation.^44^ In that analysis, we found one nominally significant SNP at the *POMC* locus, in which lower *Parasutterella* abundance was associated with the wild-type homozygous allele. Taken together, our data suggest that *Parasutterella* abundance is more related to environmental (nutrition) compared to host-genetic factors at least in the *VDR* and *POMC* regions.

Most research data regarding the role of the gut microbiome in the development of obesity and type 2 diabetes are on phylum level, showing that both diseases are associated with a reduced α- and/or β-diversity. It has to be mentioned that this reduction in diversity measures is not specific for metabolic diseases but is also found in chronic IBD,^45^ chronic heart failure^46^ and diverse oncological diseases.^47^ Thus, from our point of view, the present data and the published data on *Akkermansia* indicate that future research should also examine single bacteria or networks of defined bacteria on genus level as indicators or even pathogenic factors of specific diseases rather than solely relying on phylum data. In addition, the finding of higher *Parasutterella* abundance in obese subjects known to have lower overall diversity might underline the specificity of the novel nutrition-*Parasutterella*-host metabolic axis.

In our previous project regarding hypothalamic inflammation, we found an association of *Parasutterella* with the MRI density in the hypothalamus in severe obese subjects.^6^ This is of interest since in the present study *Parasutterella* was associated with obesity and type 2 diabetes, but not with systemic inflammatory markers like IL-6 and CRP in the periphery. As IL-6 and CRP reflect the degree of metabolic inflammation in obesity,^48^ our data might suggest that *Parasutterella* mediates its negative effects on host glucose metabolism – at least in the periphery – via direct metabolic effects rather by influencing the innate immune system. In this respect, L-cysteine, identified in our metabolomics analysis, might be an interesting candidate for future investigations.

## Conclusion

**Figure 6.**
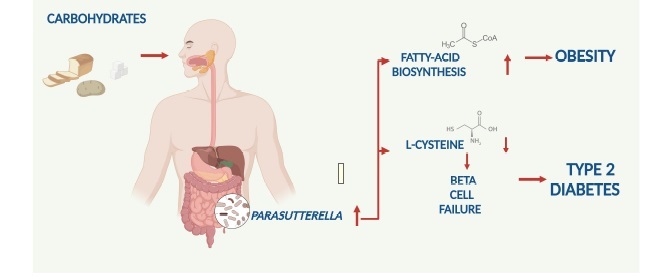
Summary figure on the proposed dietary Carbohydrate – Gut Parasutterella – Human Fatty Acid Biosynthesis metabolic axis.

Our data indicate that *Parasutterella sp*. is associated with human obesity and type 2 diabetes and might be implicated in a novel dietary carbohydrate – microbiome – host metabolic axis. Thereby, the link to the fatty acid biosynthesis pathway might be important for body weight gain in obesity in response to a carbohydrate-rich diet, whereas the link to L-cysteine could be relevant for type 2 diabetes development (Figure 6). In addition, the inverse relationship of *Parasutterella* with the dietary ω3 fatty intake should be of interest for future studies, given the convincing data of the recent REDUCE-IT trial.^49^

## Supplementary Material

Supplemental Material

## Data Availability

Samples and data are stored in the PopGen Biobank (Schleswig-Holstein, Germany) and can be accessed via a structured application procedure (https://www.uksh.de/p2n/Information+for+Researchers.html).
