# Supplementary figures and images for: A dietary carbohydrate – gut Parasutterella – human fatty acid biosynthesis metabolic axis in obesity and type 2 diabetes
Source: Gut Microbes. 2022 Apr 18;14(1):2057778. doi: 10.1080/19490976.2022.2057778 (PMC9037427; doi:10.1080/19490976.2022.2057778)

S1

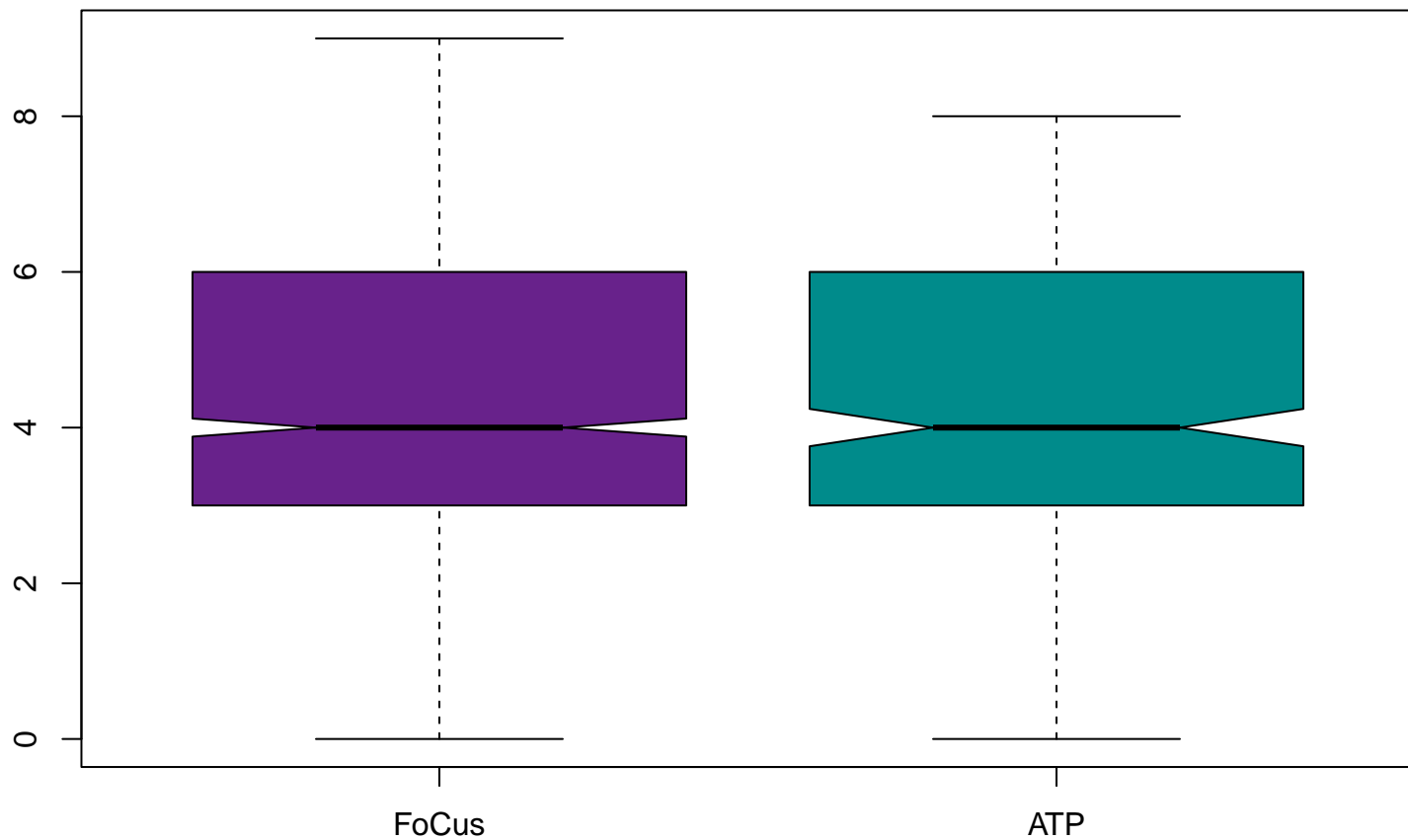

Supplement: Supplemental Material [file KGMI_A_2057778_SM9393.zip › Supp_Fig_1.pdf]

FoCus

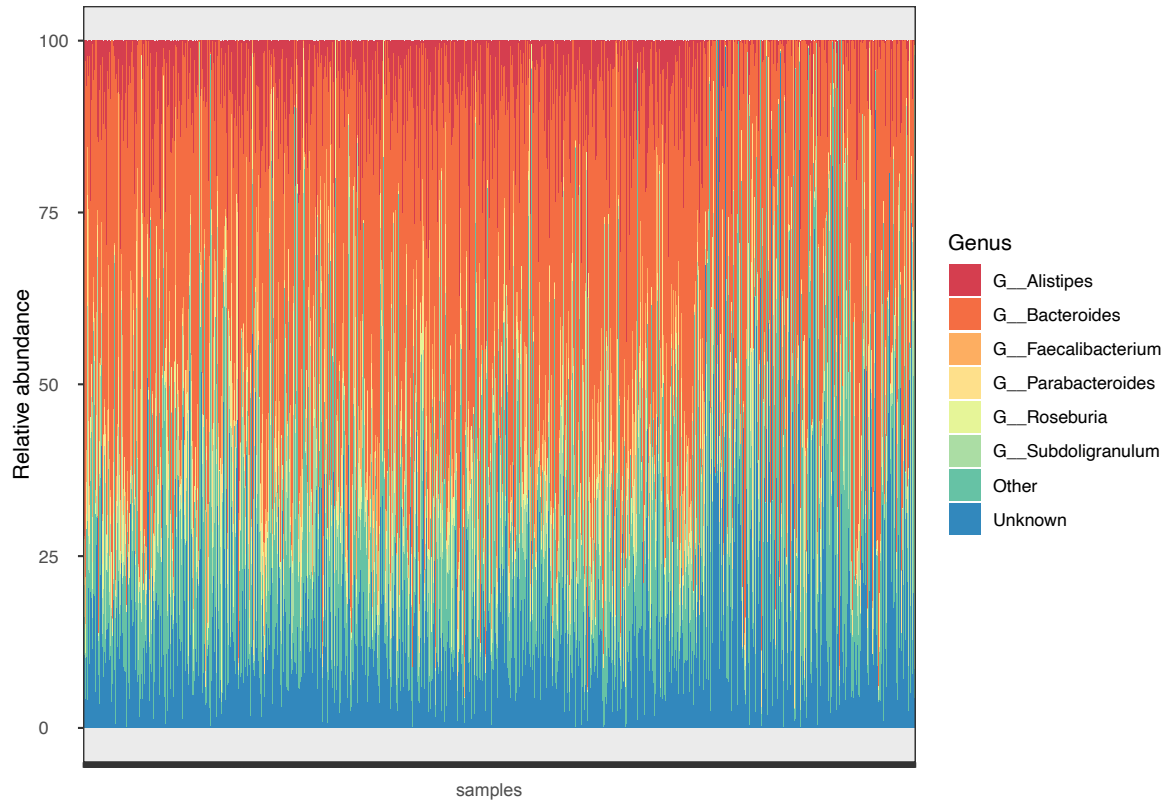

Intervention -Baseline

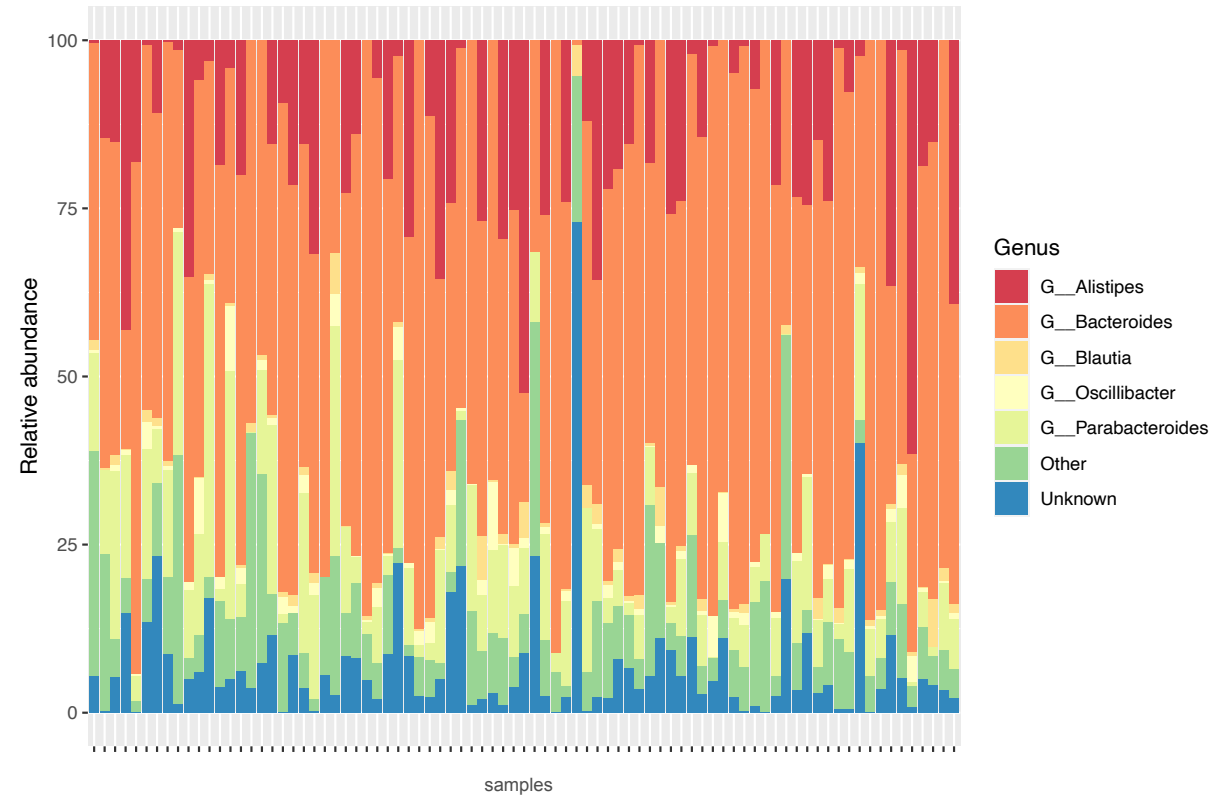

Supplement: Supplemental Material [file KGMI_A_2057778_SM9393.zip › Supp_Fig_2.pdf]
